# Risk factors for conversion to thoracotomy in patients with lung cancer undergoing video-assisted thoracoscopic surgery: A meta-analysis

**DOI:** 10.1371/journal.pone.0313236

**Published:** 2024-11-15

**Authors:** Siyu Wang, Hong Yan, Jun Wen, Zitong Zhou, Jialan Xu

**Affiliations:** School of Nursing, Chengdu University of Traditional Chinese Medicine, Chengdu, Sichuan Province, People’s Republic of China; European Institute of Oncology: Istituto Europeo di Oncologia, ITALY

## Abstract

**Objective:**

To systematically evaluate the risk factors of conversion to thoracotomy in thoracoscopic surgery (VATS) for lung cancer, and to provide a theoretical basis for the development of personalized surgical plans.

**Methods:**

CNKI, Wanfang, VIP, CBM, PubMed, Cochrane Library, Web of Science, and Embase databases were searched by computer from the establishment of the database to March 2024. Relevant studies on the risk factors of conversion to thoracotomy in VATS for lung cancer were searched. Two reviewers independently performed literature screening, data extraction, and quality evaluation, and Stata16.0 software was used for data analysis.

**Results:**

A total of 14 studies were included in this study, with a total sample size of 10605, and a total of 11 risk factors were obtained. Mate analysis showed that, Age ≥ 65 years old [*OR*(95%*CI*) = 2.61(1.67,4.09)], male [*OR*(95%*CI*) = 1.46(1.19,1.79)], BMI(Body Mass Index) ≥ 25 [*OR*(95%*CI*) = 1.79(1.17,2.74)], tuberculosis history [*OR*(95%*CI*) = 7.67(4.25,13.83)], enlarged mediastinal lymph nodes [*OR*(95%*CI*) = 2.33(1.50,3.06)], lung door swollen lymph nodes [*OR*(95%*CI*) = 6.33(2.07,19.32)], pleural adhesion [*OR*(95%*CI*) = 2.50(1.93,3.25)], tumor located in the lung Upper lobe [*OR*(95%*CI*) = 4.01(2.87,5.60)], sleeve lobectomy [*OR*(95%*CI*) = 3.40(1.43,8.08)], diameter of tumor ≥ 3.5cm [*OR*(95%*CI*) = 2.13(1.15,3.95)] associated with lung cancer VATS transit thoracotomy.

**Conclusions:**

Age ≥ 65 years old, male, BMI ≥ 25, tuberculosis history, enlarged mediastinal lymph nodes, lung door swollen lymph nodes, pleural adhesion, tumor located in the lung Upper lobe, sleeve lobectomy, diameter of tumor ≥ 3.5cm are risk factors for conversion to thoracotomy during VATS for lung cancer. Clinicians should pay attention to the above factors before VATS to avoid forced conversion due to the above factors during VATS. Due to the number and limitations of the included studies, the above conclusions need to be validated by additional high-quality studies.

**Trail registration:**

The protocol was registered into the PROSPERO database under the number CRD42023478648.

## Introduction

Lung cancer is one of the most prevalent malignant neoplastic diseases and the leading cause of cancer mortality. It is estimated that approximately two million new cases of lung cancer and 1.76 million lung cancer-related deaths occur globally each year [1]. Additionally, lung cancer has a high incidence and mortality rate in China. According to the International Agency for Research on Cancer Global Cancer Statistics, the number of new cases and deaths of lung cancer in China is about 820000 and 710000 in 2020 [2]. Surgery is the best treatment for lung cancer, and common surgical modalities include traditional thoracotomy and video-assisted thoracic surgery (VATS) [3]. Compared with traditional thoracotomy, VATS has the advantages of smaller surgical wounds, less postoperative pain, and fewer postoperative complications, and has gradually become the preferred surgical treatment for lung cancer [4]. However, lung cancer VATS requires not only the removal of cancerous lung tissue but also the dissection of surrounding lymph nodes, which is still at risk in lung cancer patients during the process of VATS due to the small surgical incision and narrow surgical field [5]. Studies have reported that the conversion rate of VATS to thoracotomy in lung cancer is 2%~23% [6]. Conversion to thoracotomy during VATS will inevitably result in an enlargement of the surgical wound, an extension of the surgical procedure and hospital stay, and an increased likelihood of a poor prognosis. At present, there are more and more studies on the risk factors of conversion to thoracotomy during VATS for lung cancer, but the results are not completely consistent. Therefore, this study aims to systematically evaluate the risk factors of conversion to thoracotomy during VATS for lung cancer by searching various databases, to help clinicians find high-risk patients as early as possible and develop personalized coping plans in advance.

## Materials and methods

### Literature search strategy

The related articles on the influencing factors of conversion to thoracotomy during VATS for lung cancer were searched by the combination of subject words and free words in CNKI, Wanfang, VIP, CBM, PubMed, Cochrane Library, Web of Science, and Embase databases. The search time limit was from the establishment of the database to March 2024. The search terms were lung cancer, pulmonary cancer, pulmonary neoplasm, video-assisted thoracic surgery, VATS, thoracoscopic Surgery, conversion to thoracotomy, and thoracotomy, retrieved articles were simultaneously screened for their references. The complete search strategy is shown in S1 File. Furthermore, databases such as ClinicalTrials.gov and the World Health Organization’s ICTRP were also searched for ongoing clinical trials.

### Literature inclusion and exclusion criteria

Inclusion Criteria: (1) The type of study was a case-control study or cohort study; (2) The subjects were lung cancer patients undergoing VATS; (3) Any study that includes risk factors for conversion to thoracotomy during VATS for lung cancer; (4) The outcome was conversion to thoracotomy during VATS for lung cancer.

Exclusion Criteria: (1) The language of the literature was not Chinese or English; (2) Case studies, reviews, conference reports, etc. (3) Repeated searches and publications; (4) Studies that did not report relevant data; (5) Inaccessible or retracted literature; (6) Patient has a combination of other thoracic cancers, such as esophageal cancer.

### Literature screening and data extraction

Two reviewers independently performed literature screening and data extraction. Firstly, the literature retrieved in different databases was exported to EndNoteX9 for literature duplication removal. The title and abstract of the literature were preliminarily screened, and the full text was downloaded and read. In case of disagreement, two reviewers could negotiate or ask a third reviewer to repeat the above process and make a judgment. The final data extracted mainly included the first author, publication year, country, surgical method, sample size, rate of conversion to thoracotomy, and risk factors.

### Literature quality assessment

The content of the literature was subjected to a detailed evaluation by two independent reviewers to ascertain the potential for bias. The quality of case-control studies and cohort studies was evaluated by the Newcastle-Ottawa Scale (NOS) [7]. The total score of the scale was 9 points, 0–3 points for low-quality literature, 4–6 points for medium-quality literature, and 7–9 points for high-quality literature. When the evaluation results were inconsistent, two reviewers could negotiate or a third literature evaluator could be asked to evaluate.

### Statistical methods

Stata16.0 was used for meta-analysis, and odds ratio (OR) and 95% confidence interval (CI) were used to combine the effect sizes. The heterogeneity test was analyzed by Q test and I^2^ quantification. If I^2^≤50% and P≥0.1, the heterogeneity of each included literature was small, and the fixed effect model was used to combine the effect size. If I^2^>50% and P<0.1, the heterogeneity of each included literature was large, and the random effect model was used to combine the effect size. The test level of the combined effect size was set as α = 0.05. Sensitivity analysis was performed using Stata software. A funnel plot was used to detect publication bias when the number of included studies was more than 10.

## Result

### Results of the literature search

**Fig 1:**
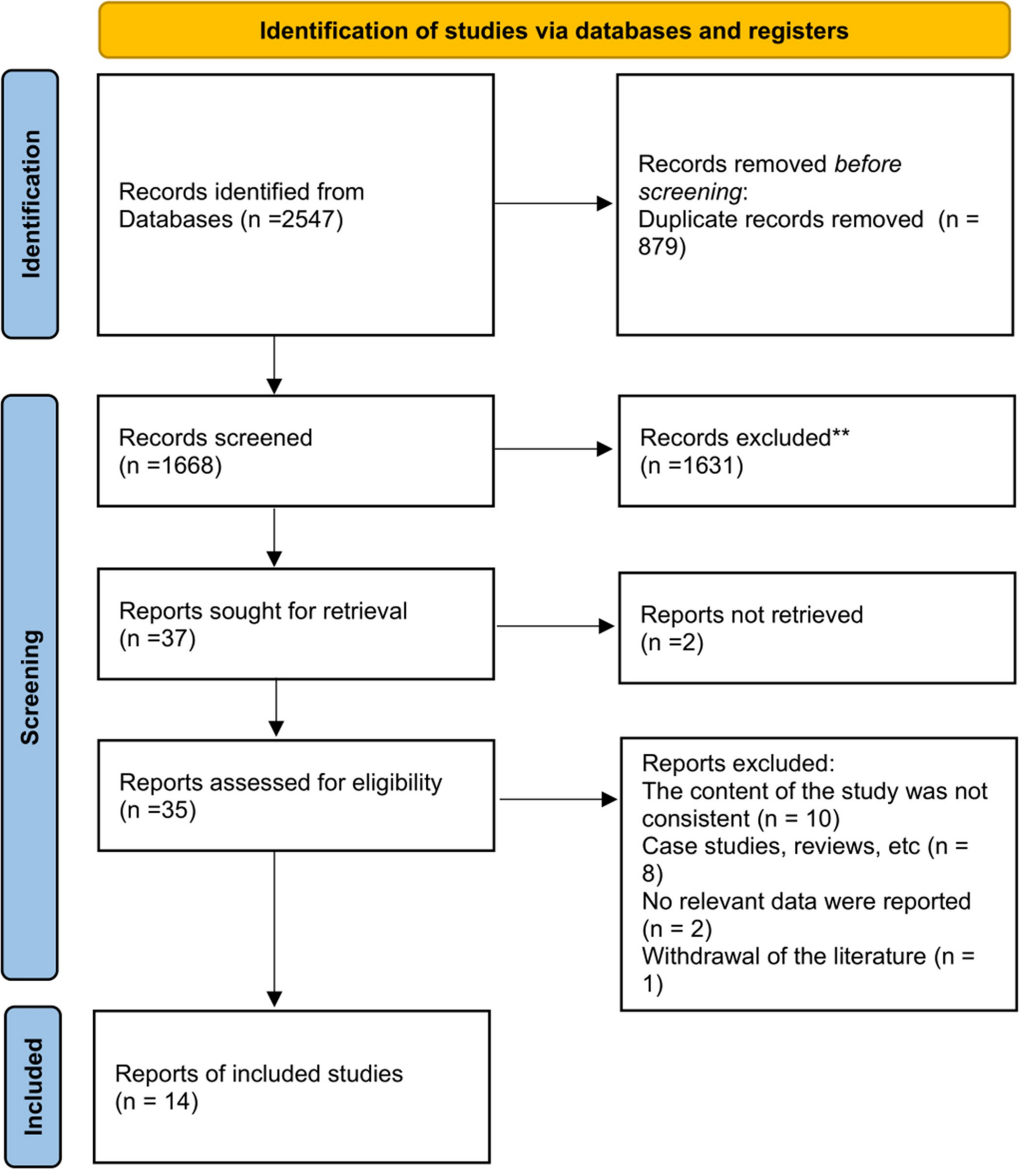
Flow chart of literature screening.

A total of 2547 relevant literature were retrieved from the databases, including 1477 Chinese literature and 1070 English literature. No ongoing studies or other unpublished literature were identified during the course of this investigation. A total of 14 articles were included after literature duplication removal, primary screening, and secondary screening. The literature screening flow chart is shown in Fig 1.

### Basic information of the included literature

All the included studies were case-control studies, including 7 Chinese articles and 7 English articles, involving a total of 11 risk factors. The lowest quality score of the literature was 6 points and the highest was 8 points. Table 1 shows the basic information of the included literature. The particular NOS score attributed to each study is presented in S1 Data.

**Table 1:** Basic information table of included literature.

Included literature	Country	Surgical methods	Sample size		Rate of conversion to thoracotomy	Risk factors	Quality score
			Thoracoscopy group	Transferred thoracotomy group			
Jing Liu 2022 [8]	China	VATS radical lobectomy	420	43	9.3%	a, d, e, f, h, g	7
Xin Liu 2023 [9]	China	VATS lobectomy	96	24	20.0%	a, g, j	6
Fu-Qian Wu 2020 [10]	China	VATS radical lobectomy	146	80	35.4%	a, d, g, h	8
Jie-Sheng Zhou 2023 [11]	China	VATS radical resection of lung cancer	470	120	20.3%	d, g, h, i	8
Wei-Hua Li 2017 [12]	China	VATS lobectomy	378	45	10.6%	e, f, i	7
Hai Li 2018 [13]	China	VATS radical lobectomy	117	65	35.7%	a, b, g, h, j	7
An- Ge Chen 2021 [14]	China	VATS radical resection of lung cancer	97	29	23.0%	a, d, g, h	6
Lim 2017 [15]	South Korea	VATS lobectomy	180	55	23.4%	a	6
Kim 2017 [16]	South Korea	VATS lobectomy and segmental resection	51	38	42.7%	a, h	6
Li 2017 [17]	China	VATS Single lobectomy	459	16	3.4%	a, c, d, g	6
Gabryel 2021 [18]	Poland	VATS anatomical segmentectomy	897	105	10.5%	e, g	7
Chen 2021 [19]	China	VATS anatomical segmentectomy	997	180	15.3%	c, g, j	6
Bongiolatti 2019 [20]	Italy	VATS lobectomy	4197	432	9.3%	a, b, k	7
Fourdrain 2022 [21]	France	VATS anatomical segmentectomy	7100	843	10.6%	c, i, k	8

Note: “a” is age, “b” is gender, “c” is BMI, “d” is history of pulmonary tuberculosis, “e” is mediastinal lymph node enlargement, “f” is hilar lymph node enlargement, “g” is pleural adhesion, “h” is tumor location, “I” is sleeve lobectomy, “j” is tumor diameter, “k” is clinical stage

### Results of meta-analysis

The results of the meta-analysis for each risk factor are presented in Table 2, while forest plots for each risk factor are provided in S1 and S2 Figs. The particular data extracted for each study are presented in S2 Data.

**Table 2:** Results of the meta-analysis.

Risk Factors	Number of Included studies	Results of heterogeneity test		Model of effect	*OR* (95%*CI*)	*P* value for the total effect
		*P*	*I* ^*2*^			
Age ≥ 65	5	0.116	46%	Fixed	2.61 [1.67, 4.09]	<0.001
Male	2	0.962	0%	Fixed	1.46 [1.19, 1.79]	<0.001
BMI≥25	2	0.556	0%	Fixed	1.79 [1.17, 2.74]	0.008
History of tuberculosis	4	0.301	18%	Fixed	7.67 [4.25, 13.83]	<0.001
Mediastinal lymph node enlargement	3	0.312	14%	Fixed	2.33 [1.50, 3.06]	<0.001
Swollen hilar lymph nodes	2	0.153	51%	Random	6.33 [2.07, 19.32]	0.001
Pleural adhesions	9	0.235	24%	Fixed	2.80 [2.27, 3.46]	<0.001
The tumor is located in the upper lobe of the lung	7	0.305	16%	Fixed	4.01 [2.87, 5.60]	<0.001
Sleeve lobectomy	2	0.106	62%	Random	3.40 [1.43, 8.08]	<0.001
Tumor diameter ≥3.5cm	2	0.998	0%	Fixed	2.13 [1.15, 3.95]	0.016
TNM clinical stage Ⅰ	2	0.002	89%	Random	1.42 [0.68, 2.97]	0.352

Note: “cm” is centimeter; “TNM clinical stage Ⅰ” is lung cancer cells located in the primary site without metastasis

### Patient’s risk factors

The patient’s risk factors included age, gender, BMI, and history of tuberculosis. Eight studies [8–10, 13–17] reported on the association between age and conversion to thoracotomy in VATS for lung cancer, of which five studies [8, 9, 13, 15, 17] had consistent criteria and could be combined. The combined results of fixed-effects models indicated that the risk of conversion to thoracotomy in patients aged ≥65 years was 2.61 times higher than that in patients aged <65 years. The combined results of fixed-effects models from 2 studies [13, 20] showed that the risk of conversion to thoracotomy during VATS in male patients was 1.46 times higher than that in females. The association between BMI and mid-stage open chest in VATS for lung cancer was reported in three studies [17, 19, 21] of which two studies [17, 19] had consistent delineation criteria and could be combined. The combined fixed-effects model demonstrated that patients with a BMI≥25 exhibited a 1.79-fold increased risk of conversion to thoracotomy during VATS compared to those with a BMI < 25. Five studies [8, 10, 11, 14, 17] reported on the association between a history of tuberculosis and conversion to thoracotomy during VATS for lung cancer. Following sensitivity analyses, four of these studies [8, 10, 14, 17] could be combined for a meta-analysis. The combined results of the fixed-effects models demonstrated that patients with a history of tuberculosis exhibited a 7.67-fold increased risk of conversion to thoracotomy during VATS in comparison with those without a history of tuberculosis.

#### Risk factors for peri-tumor tissues

Risk factors for peri-tumor tissues include enlarged mediastinal lymph nodes, enlarged hilar lymph nodes, and pleural adhesions. The findings from the fixed-effects models of three studies [8, 12, 18] demonstrated that individuals exhibiting mediastinal lymph node enlargement exhibited a 2.33-fold elevated risk of conversion to thoracotomy during VATS for lung cancer compared to those without mediastinal lymph node enlargement. The combined random-effects model results from two studies [8, 12] demonstrate that the risk of thoracotomy during VATS for lung cancer is 6.33 times greater for patients with hilar lymph node enlargement compared to those without. The combined fixed-effects model results of nine studies [8–11, 13, 14, 17–19] demonstrated that patients with pleural adhesions exhibited a 2.80-fold increased risk of conversion to thoracotomy during VATS for lung cancer compared to those without pleural adhesions. After correction by the cut-and-patch method, the result was 2.5 times higher.

#### Risk factors for tumor characteristics and mode of resection

Risk factors for Tumor characteristics and resection modalities included Tumor location, sleeve lobectomy, Tumor diameter, and tumor clinical stage. The aggregated results of the fixed-effects model of the seven studies [8–11, 13, 14, 16] demonstrated that patients with Tumors situated in the upper lobes of the lung exhibited a 4.10-fold elevated risk of conversion to thoracotomy during VATS for lung cancer in comparison to those with Tumors in the non-upper lobes of the lung. The combined random-effects model results of the two studies [11, 12] demonstrated that patients undergoing sleeve lobectomy exhibited a 4.10-fold increased risk of conversion to thoracotomy during VATS compared to lobectomy. Three studies [9, 13, 19] reported on the association between Tumor diameter and intermediate chest opening in VATS for lung cancer. Of these, two studies [9, 13] had consistent delineation criteria and could be combined. The combined results of the fixed-effects model demonstrated that patients with Tumor diameters≥3.5 cm exhibited a 2.13-fold increased risk of conversion to thoracotomy during VATS compared to those with diameters < 3.5 cm. The combined results of the random-effects models of the two studies [20, 21] demonstrated no statistically significant difference in the effect of Tumor clinical stage on the conversion to thoracotomy during VATS for lung cancer.

#### Sensitivity analysis and publication bias

**Fig 2:**
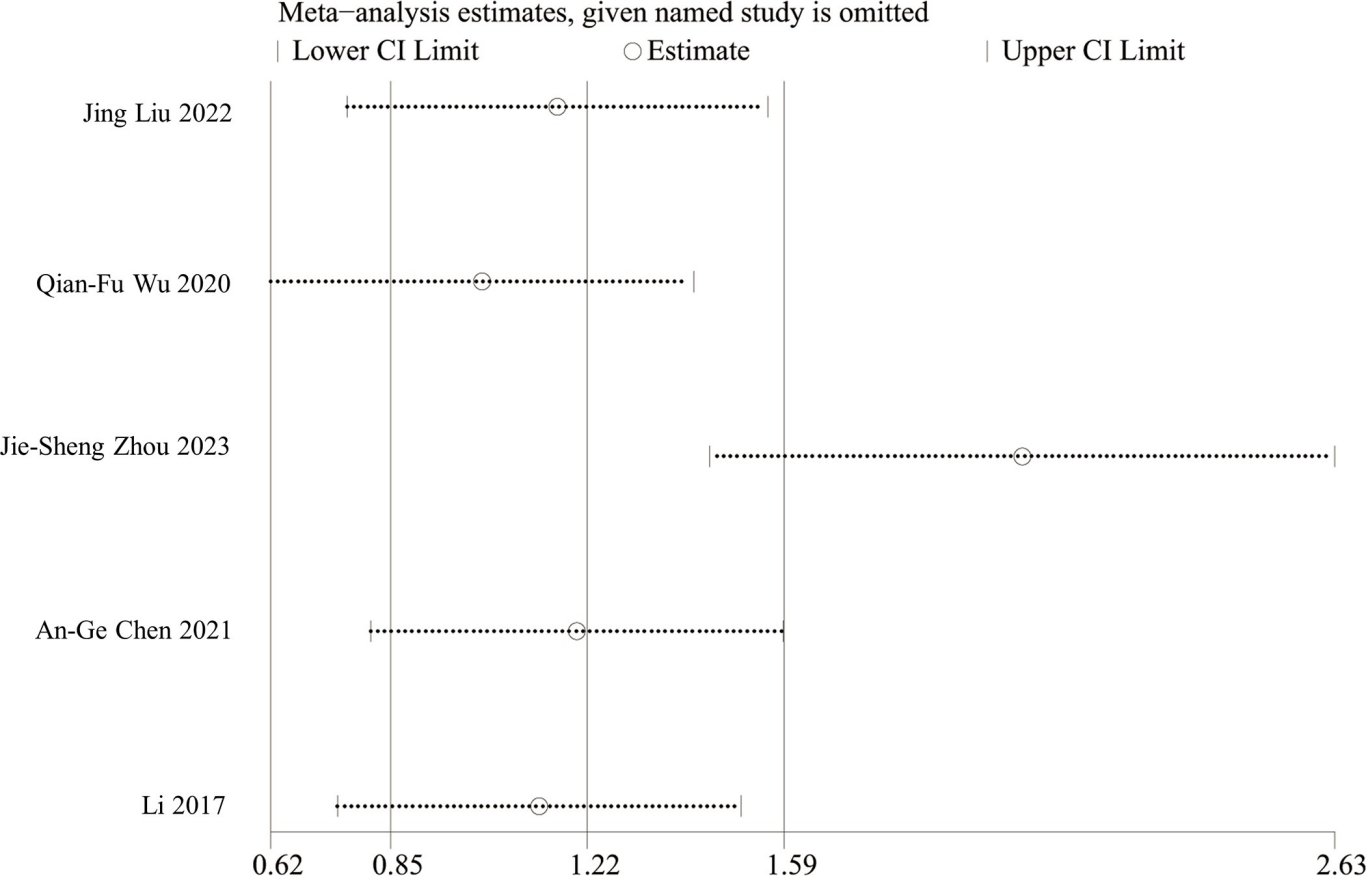
Sensitivity analysis of pulmonary tuberculosis history.

A sensitivity analysis was conducted for each risk factor using the one-by-one exclusion method and the interconversion of fixed-effects and random-effects models. The results of the one-by-one exclusion method indicated that there were some discrepancies between the study conducted by Zhou [11] and other studies examining the history of tuberculosis. After the exclusion, the I^2^ value decreased from 75% to 18%. The sensitivity analysis of the history of tuberculosis is shown in Fig 2. The findings of the model transformation method demonstrated that the results of clinical staging stage I exhibited significant variability across different models, indicating a potential for unreliable outcomes. The sensitivity analysis results of the other factors were relatively stable. The Egger test indicated the possibility of publication bias in the pleural adhesion factor (P≤0.05), and the effect sizes were adjusted through the use of the cut-and-patch method. The adjusted OR was found to be 2.50 (95% CI: 1.93–3.25), demonstrating no significant change compared to the pre-adjustment period. This finding suggests that the publication bias did not exert a notable impact on the meta-analysis results. Consequently, the results obtained were deemed to be stable. Additionally, the remaining risk factors did not exhibit any substantial publication bias (P>0.05). The sensitivity analyses and publication bias for each factor are shown in Table 3.

**Table 3:** Sensitivity analyses and publication bias results.

Risk Factors	fixed-effects model		random-effects model		Egger test
	*OR* (95%*CI*)	*P*值	*OR* (95%*CI*)	*P*值	
Age ≥ 65	2.61 [1.67, 4.09]	<0.001	2.73 [1.47, 5.09]	0.002	0.418
Male	1.46 [1.19, 1.79]	<0.001	1.46 [1.19, 1.79]	<0.001	—
BMI≥25	1.79 [1.17, 2.74]	0.008	1.79 [1.17, 2.74]	0.008	—
History of tuberculosis	7.67 [4.25, 13.83]	<0.001	7.72 [3.99, 14.93]	<0.001	0.648
Mediastinal lymph node enlargement	2.33 [1.50, 3.06]	<0.001	2.30 [1.43, 3.71]	0.001	0.653
Swollen hilar lymph nodes	6.04 [2.78, 13.12]	<0.001	6.33 [2.07, 19.32]	0.001	—
Pleural adhesions	2.80 [2.27, 3.46]	<0.001	2.91 [2.26 3.74]	<0.001	0.019
The tumor is located in the upper lobe of the lung	4.01 [2.87, 5.60]	<0.001	4.10 [2.81, 5.96]	<0.001	0.443
Sleeve lobectomy	3.06 [1.86, 5.04]	0.001	3.40 [1.43, 8.08]	<0.001	—
Tumor diameter ≥3.5cm	2.13 [1.15, 3.95]	0.016	2.13 [1.15, 3.95]	0.016	—
TNM clinical stage Ⅰ	1.60 [1.27, 2.00]	<0.001	1.42 [0.68, 2.97]	0.352	—

Note: “—” is result not shown

## Discussion

With the ongoing advancement of thoracoscopic surgery, the incidence of conversion to thoracotomy during VATS for lung cancer has declined since 2016 [22]. However, some lung cancer patients still require conversion to thoracotomy intraoperatively, which significantly increases the complexity of the procedure and the surgical trauma, and has a profound impact on the patient’s postoperative recovery [23]. It is therefore essential to select the appropriate surgical technique by the risk factors associated with VATS.

### Impact of patient’s factors on conversion to thoracotomy during VATS for lung cancer

Some studies have found that thoracoscopic lung surgery requires longer operation time than open chest surgery [24], and patients aged 65 years or above exhibit a significantly shorter operative tolerance period due to the effects of advanced age, which increases the risk of undergoing intermediate thoracotomy due to their inability to tolerate the procedure for an extended period. VATS surgery exceeding 360 minutes may elevate the likelihood of postoperative complications. Therefore, VATS in older patients should be expedited to completion within 360 minutes if feasible.

Men are the main group of smokers [25]. Long-term smoking exposes lung tissue to the chemical substances produced by tobacco repeatedly, which causes inflammatory reactions and cell damage, leading to a more complicated surgical process [26], and the risk of conversion to thoracotomy is greater. A study demonstrated that non-smoking resulted in a reduction in intraoperative blood loss; however, the length of time spent abstaining from smoking did not affect intraoperative blood loss [27]. Furthermore, a reduction in intraoperative blood loss lowers the probability of conversion to a thoracotomy procedure during VATS. Consequently, it is recommended that surgeons request patients to abstain from smoking during the perioperative period, even in the absence of cessation after the initial diagnosis of the Tumor. This will have a beneficial impact on the surgical procedure and the postoperative recovery period [28].

During thoracoscopic surgery, overweight or obese patients with BMI≥25 are more likely to develop atelectasis and hypoxemia due to the accumulation of subcutaneous fat, which leads to limited diaphragmatic movement and thoracic expansion, and decreased lung compliance [29]. To reduce adverse symptoms, they are forced to convert to thoracotomy. When dealing with overweight or obese patients with a BMI ≥25, the surgeon should develop a personalized surgical plan based on the patient’s weight.

Studies have found [30] that pulmonary tuberculosis can cause severe calcification and adhesion of lymph nodes, which seriously interferes with the visual field under thoracoscopic surgery. To expand the surgical field, conversion to thoracotomy is necessary. Accordingly, when encountering patients with a history of tuberculosis, it is imperative to conduct a meticulous assessment of lymph node calcification and adhesions before selecting an optimal surgical procedure.

### The influence of surrounding tissues on conversion to thoracotomy during VATS for lung cancer

Lymph nodes are generally distributed along the direction of blood vessels. When various factors lead to the enlargement of mediastinal and hilar lymph nodes, the clarity of local anatomical structures will be affected, causing serious interference with the field of view under thoracoscopic surgery and increasing the risk of vascular injuries, which will lead to the conversion to thoracotomy procedure during VATS for lung cancer [31]. According to the study of Matsuoka et al. [32], the conversion rate of thoracoscopic surgery to thoracotomy due to pleural adhesion was about 8%-20%. It is difficult to treat patients with pleural adhesion under thoracoscopy, which can easily lead to massive bleeding when separating the adhesion tissue, and the risk of conversion to thoracotomy is higher [33]. The most common cause of conversion to thoracotomy procedure during VATS is vascular injury, followed by anatomic causes such as adhesions and the presence of large or sticky lymph nodes. Before surgery, the surgeon should perform a careful assessment of the difficulty of VATS surgery in a patient based on imaging, to determine whether open chest surgery should be performed.

### The impact of tumor characteristics and resection methods on conversion to thoracotomy in VATS for lung cancer

Tumor diameter is an important indicator for choosing surgical methods, but the exact value is still unclear. It is generally believed that VTAS can be chosen when the Tumor diameter is small, and thoracotomy is chosen when it is large [34]. In this study, a Tumor diameter of 3.5 cm is considered to be the threshold for thoracotomy. For lung cancer patients with Tumors greater than or equal to 3.5 cm, the decision to perform open thoracotomy should be made with careful consideration.

It has been determined that the arterial vasculature in the upper lobes of the lungs is characterized by increased thickness, branching complexity, and the presence of intricate surrounding tissue, which collectively renders the surgical excision of this region susceptible to the potential for vascular damage [35]. The surgical procedure, termed sleeve lobectomy, has been designed to facilitate the conservation of the greatest possible amount of native lung tissue; nevertheless, the inherent technical complexity of the operation may potentially result in inadvertent damage to adjacent tissues and blood vessels [36]. Such factors increase the probability of conversion to thoracotomy during the operative period.

The impact of clinical staging on conversion to thoracotomy during VATS for lung cancer has been inconsistent across different studies. While Bongiolatti’s [20] study demonstrated no statistically significant association between staging and conversion, Fourdrain’s [21] study yielded contrasting results. The meta-analysis of the aggregated data revealed that the discrepancy in the impact of the clinical stage on the likelihood of conversion to open chest surgery in VATS for lung cancer was not statistically significant. Given that the Fourdrain study had an elevated NOS score, we believe that further investigation is warranted in this area. We expect that further high-quality literature will corroborate this view.

### Limitations and prospects

First of all, the sample size of each included literature is quite different, and the surgical methods of each study are also different, which may cause some heterogeneity. Secondly, in this study, different included literature may have different classification criteria for a factor, and some unpublished literature may not be included, resulting in a small number of included literature for some factors, and the publication bias detection cannot be completed, which may have a certain impact on the accuracy of the results. Finally, the types of studies included in this study were case-control studies, and more prospective, multi-center, and high-quality literature should be included for further verification and to obtain more risk factors of conversion to thoracotomy after VATS for lung cancer.

## Supporting information

S1 File Literature search strategy. (DOCX)

S1 Data Newcastle-Ottawa Scale score. (XLSX)

S2 Data Detailed data in the literature. (XLSX)

S3 Data Literature search results. (XLSX)

S1 Fig Forest plots of risk factors. (TIF)

S2 Fig Forest plots of risk factors. (TIF)

## References

[1] Thai AA, Solomon BJ, Sequist LV, Gainor JF, Heist RS. Lung cancer. Lancet (London, England). 2021;398(10299):535–54. Epub 2021/07/18. doi: 10.1016/S0140-6736(21)00312-3 . 34273294

[2] Cao W, Chen H-D, Yu Y-W, Li N, Chen W-Q. Changing profiles of cancer burden worldwide and in China: a secondary analysis of the global cancer statistics 2020. Chinese medical journal. 2021;134(07):783–91. doi: 10.1097/CM9.0000000000001474 33734139 PMC8104205

[3] Bouabdallah I, Pauly V, Viprey M, Orleans V, Fond G, Auquier P, et al. Unplanned readmission and survival after video-assisted thoracic surgery and open thoracotomy in patients with non-small-cell lung cancer: a 12-month nationwide cohort study. European Journal of Cardio-Thoracic Surgery. 2021;59(5):987–95. doi: 10.1093/ejcts/ezaa421 33236091

[4] Dong D, Han D-P, Han W, Chen K, Jie X, Che J-M, et al. Chinese expert consensus on the uniportal video-assisted thoracoscopic surgery for lung cancer: An interpretation Chinese Journal of Clinical Thoracic and Cardiovascular Surgery. Chinese Journal of Clinical Thoracic and Cardiovascular Surgery. 2021;28(02):137–45. doi: 10.7507/1007-4848.202011024

[5] Su P, Wen S-W, Wang M-B, Xu Y-Z, Lv H-L, Li Z-H, et al. Reasons for Conversion to Thoracotomy in 83 Cases during Video-assisted Thoracic Surgery Lobectomy: A Summary of 1,350 Consecutive Operations by A Single Surgical Team. Chinese Journal of Lung Cancer. 2021;24(07):475–82. doi: 10.3779/j.issn.1009-3419.2021.101.21 34134186 PMC8317091

[6] Sezen CB, Bilen S, Kalafat CE, Cansever L, Sonmezoglu Y, Kilimci U, et al. Unexpected conversion to thoracotomy during thoracoscopic lobectomy: a single-center analysis. General thoracic and cardiovascular surgery. 2019;67(11):969–75. Epub 2019/04/21. doi: 10.1007/s11748-019-01127-1 . 31004316

[7] Stang A. Critical evaluation of the Newcastle-Ottawa scale for the assessment of the quality of nonrandomized studies in meta-analyses. European journal of epidemiology. 2010;25(9):603–5. Epub 2010/07/24. doi: 10.1007/s10654-010-9491-z . 20652370

[8] Liu J, Zhou Y-R, Liu Y, Chen H, YU D-M. Establishment and Validation of the Prediction Model for Conversion from Thoracoscopic Lobectomy to Thoracotomy for Non-Small Cell Lung Cancer. Journal of Cancer Control And Treatment. 2022;35(12):1061–9. doi: 10.3969/j.issn.1674-0904.2022.12.001

[9] Liu X, Pei Y-J, Ren J-W. Study on High-Risk Factors of Conversion from Thoracoscopic Lobectomy to Thoracotomy in Patients with Lung Cancer. The Practical Journal of Cancer. 2023;38(4):601–3. doi: 10.3969/j.issn.1001-5930.2023.04.020

[10] Wu Q-F, Li J-H, Zhang Z-D, Lin Z-H, Wei H-L, Peng w. Influencing factors of conversion to thoracotomy in elderly patients undergoing totally thoracoscopic radical resection of lung cancer. Chinese Journal of Gerontology. 2020;40(12):2536–9. doi: 10.3969/j.issn.1005-9202.2020.12.023

[11] Zhou S-J, Zhou H, Hu Q, Lin Y-D, Yang Y. Risk factors of conversion from thoracoscopic radical resection to thoracotomy for lung cancer. Journal of Clinical Pulmonary Medicine. 2023;28(5):724–7. doi: 10.3969/j.issn.1009-6663.2023.05.016

[12] Li H-W, Wang H-Y, Zhang L-Y. Analysis of risk factors for conversion to thoracotomy during video-assisted thoracic surgery lobectomy for lung cancer. Chinese Journal of Clinical Thoracic and Cardiovascular Surgery. 2017;24(12):962–9. doi: 10.7507/1007-4848.201609064

[13] Li H, Xu G, Song Y-X, Cai Q-Y, Li J, Chen C. Risk factor analysis of video-assisted thoracoscopic lobectomy for conversion to thoracotomy surgery in patients with non-small-cell lung cancer. Acta Universitatis Medicinalis Anhui. 2018;53(5):809–11. doi: 10.19405/j.cnki.issn1000-1492.2018.05.032

[14] Chen A-G, Zhao Q-F. Analysis of Influencing Factors of Patients Undergoing Thoracoscopic Radical Resection of Lung Cancer Turning to Thoracotomy. Journal of Mathematical Medicine. 2021;34(3):379–82. doi: 10.3969/j.issn.1004-4337.2021.03.029

[15] Lim CG, Shin KM, Lim JS, Lim JK, Kim HJ, Kim WH, et al. Predictors of conversion to thoracotomy during video-assisted thoracoscopic surgery lobectomy in lung cancer: additional predictive value of FDG-PET/CT in a tuberculosis endemic region. Journal of thoracic disease. 2017;9(8):2427–36. Epub 2017/09/22. doi: 10.21037/jtd.2017.07.40 ; PubMed Central PMCID: PMC5594172. 28932548 PMC5594172

[16] Kim SW, Hong JM, Kim D. What is difficult about doing video-assisted thoracic surgery (VATS)? A retrospective study comparing VATS anatomical resection and conversion to thoracotomy for lung cancer in a university-based hospital. Journal of thoracic disease. 2017;9(10):3825–31. Epub 2017/12/23. doi: 10.21037/jtd.2017.09.98 ; PubMed Central PMCID: PMC5723815. 29268391 PMC5723815

[17] Li SJ, Zhou K, Shen C, Li PF, Wu YM, Wang ZQ, et al. Body surface area: a novel predictor for conversion to thoracotomy in patients undergoing video-assisted thoracoscopic lung cancer lobectomy. Journal of thoracic disease. 2017;9(8):2383–96. Epub 2017/09/22. doi: 10.21037/jtd.2017.07.53 ; PubMed Central PMCID: PMC5594169. 28932543 PMC5594169

[18] Gabryel P, Piwkowski C, Kasprzyk M, Zieliński P, Roszak M, Dyszkiewicz W. Worse outcomes after conversion of thoracoscopic lobectomy for lung cancer. Interactive cardiovascular and thoracic surgery. 2021;32(3):356–63. Epub 2020/11/23. doi: 10.1093/icvts/ivaa274 ; PubMed Central PMCID: PMC8906676. 33221893 PMC8906676

[19] Chen D, Kang P, Tao S, Wu L, Li Q, Tan Q. Risk factors of conversion in robotic- and video-assisted pulmonary surgery for non-small cell lung cancer. Updates in surgery. 2021;73(4):1549–58. Epub 2021/01/06. doi: 10.1007/s13304-020-00954-9 . 33398772

[20] Bongiolatti S, Gonfiotti A, Viggiano D, Borgianni S, Politi L, Crisci R, et al. Risk factors and impact of conversion from VATS to open lobectomy: analysis from a national database. Surgical endoscopy. 2019;33(12):3953–62. Epub 2019/02/02. doi: 10.1007/s00464-019-06682-5 . 30706153

[21] Fourdrain A, Georges O, Gossot D, Falcoz PE, Jougon J, Baste JM, et al. Patient risk factors for conversion during video-assisted thoracic surgery Epithor conversion score. European Journal of cardio-thoracic Surgery: official journal of the European Association for Cardio-thoracic Surgery. 2022;62(3). Epub 2022/04/24. doi: 10.1093/ejcts/ezac249 . 35459942

[22] Tong C, Li T, Huang C, Ji C, Liu Y, Wu J, et al. Risk Factors and Impact of Conversion to Thoracotomy From 20,565 Cases of Thoracoscopic Lung Surgery. The Annals of thoracic surgery. 2020;109(5):1522–9. Epub 2020/01/26. doi: 10.1016/j.athoracsur.2019.12.009 . 31981504

[23] Bourdages-Pageau E, Vieira A, Lacasse Y, Figueroa PU, editors. Outcomes of uniportal vs multiportal video-assisted thoracoscopic lobectomy. Seminars in thoracic and cardiovascular surgery; 2020: Elsevier. 10.1053/j.semtcvs.2019.05.021 31150825

[24] Battoo A, Jahan A, Yang Z, Nwogu CE, Yendamuri SS, Dexter EU, et al. Thoracoscopic pneumonectomy: an 11-year experience. Chest. 2014;146(5):1300–9. Epub 2014/08/03. doi: 10.1378/chest.14-0058 . 25086234

[25] Wu L-L, Han R-Q, Liu F-D, Feng-Yuan-Yuan. Survival analysis of cancer between 2012 and 2020 in Yancheng City. Chinese Journal of Cancer Prevention and Treatment. 2023;30(10):571–6,86. doi: 10.16073/j.cnki.cjcpt.2023.10.01

[26] Wei J-J, Wei J-S. Bilirubin mitigates lung tissue damage due to smoking exposure by influencing macrophage efferocytosis. Current Immunology. 2023;43(4):301–6,11.

[27] Xu LM, Dai SP, Zuo YX. Impacts of Preoperative Smoking and Smoking Cessation Time on Preoperative Peripheral Blood Inflammatory Indexes and Postoperative Hospitalization Outcome in Male Patients with Lung Cancer and Surgery Treatment. Chinese medical sciences journal = Chung-kuo i hsueh k’o hsueh tsa chih. 2020;35(2):170–8. Epub 2020/07/21. doi: 10.24920/003540 . 32684237

[28] Phillips JD, Fay KA, Ramkumar N, Hasson RM, Fannin AV, Millington TM, et al. Long-Term Outcomes of a Preoperative Lung Resection Smoking Cessation Program. The Journal of Surgical Research. 2020;254:110–7. Epub 2020/05/20. doi: 10.1016/j.jss.2020.04.005 ; PubMed Central PMCID: PMC10750226. 32428728 PMC10750226

[29] Leonardi B, Forte S, Natale G, Messina G, Rainone A, Opromolla G, et al. One-lung ventilation in obese patients undergoing thoracoscopic lobectomy for lung cancer. Thoracic cancer. 2023;14(3):281–8. Epub 2022/12/09. doi: 10.1111/1759-7714.14747 ; PubMed Central PMCID: PMC9870737. 36479830 PMC9870737

[30] Wen L-W, Hou D-L. Current status and progress of imaging evaluation methods for detecting pulmonary tuberculosis combined with lung cancer. Chinese Journal of Antituberculosis. 2023;45(6):620–4. doi: 10.19982/j.issn.1000-6621.20230045

[31] Muslim Z, Stroever S, Poulikidis K, Weber JF, Connery CP, Herrera LJ, et al. Conversion to Thoracotomy in Non-Small Cell Lung Cancer: Risk Factors and Perioperative Outcomes. Innovations (Philadelphia, Pa). 2022;17(2):148–55. Epub 2022/05/03. doi: 10.1177/15569845221091979 . 35499922

[32] Matsuoka K, Yamada T, Matsuoka T, Nagai S, Ueda M, Miyamoto Y. Analysis of conversion to thoracotomy during thoracoscopic lung resection. Asian cardiovascular & thoracic annals. 2019;27(5):381–7. Epub 2019/05/11. doi: 10.1177/0218492319851396 . 31072106

[33] Kobayashi N, Kawamura T, Yanagihara T, Saeki Y, Kikuchi S, Goto Y, et al. [Influence of Pleural Adhesions on Thoracoscopic Surgeries for Malignant Lung Tumors]. Kyobu geka The Japanese Journal of thoracic surgery. 2021;74(7):509–13. Epub 2021/07/02. . 34193785

[34] Zhou SJ, Pei GT, Han Y, Yu DP, Song XY, Li YS, et al. Sleeve lobectomy by video-assisted thoracic surgery versus thoracotomy for non-small cell lung cancer. JOURNAL OF CARDIOTHORACIC SURGERY. 2015;10. doi: 10.1186/s13019-015-0318-6 WOS:000360963100001. 26357875 PMC4564953

[35] Pan Q, Zuo C-T, Mao N-Q, Wang S-F, Wu J-W. Clinical effect of video-assisted thoracoscopic anatomical pneumonectomy for stage I lung cancer. Journal of Clinical and Experimental Medicine. 2020;19(8):870–3. doi: 10.3969/j.issn.1671-4695.2020.08.025

[36] Pischik VG. Technical difficulties and extending the indications for VATS lobectomy. Journal of thoracic disease. 2014;6:S623–S30. doi: 10.3978/j.issn.2072-1439.2014.10.11 WOS:000347239700005. 25379200 PMC4221338

