# Supplementary material for: Risk factors for conversion to thoracotomy in patients with lung cancer undergoing video-assisted thoracoscopic surgery: A meta-analysis
Source: PLoS One. 2024 Nov 15;19(11):e0313236. doi: 10.1371/journal.pone.0313236 (PMC11567592; doi:10.1371/journal.pone.0313236)
Supplement: S1 File — (DOCX) [file pone.0313236.s001.docx]

**Search strategy-pubmed**

("thoracic surgery, video assisted"[MeSH Terms] OR ("thoracic"[All Fields] AND "surgery"[All Fields] AND "video assisted"[All Fields]) OR "video-assisted thoracic surgery"[All Fields] OR ("video"[All Fields] AND "assisted"[All Fields] AND "thoracic"[All Fields] AND "surgery"[All Fields]) OR "video assisted thoracic surgery"[All Fields] OR ("thoracic surgery, video assisted"[MeSH Terms] OR ("thoracic"[All Fields] AND "surgery"[All Fields] AND "video assisted"[All Fields]) OR "video-assisted thoracic surgery"[All Fields] OR "vats"[All Fields])) AND ("lung neoplasms"[MeSH Terms] OR ("lung"[All Fields] AND "neoplasms"[All Fields]) OR "lung neoplasms"[All Fields] OR ("lung neoplasms"[MeSH Terms] OR ("lung"[All Fields] AND "neoplasms"[All Fields]) OR "lung neoplasms"[All Fields] OR ("pulmonary"[All Fields] AND "neoplasm"[All Fields]) OR "pulmonary neoplasm"[All Fields]) OR ("lung neoplasms"[MeSH Terms] OR ("lung"[All Fields] AND "neoplasms"[All Fields]) OR "lung neoplasms"[All Fields] OR ("lung"[All Fields] AND "cancer"[All Fields]) OR "lung cancer"[All Fields]) OR ("lung neoplasms"[MeSH Terms] OR ("lung"[All Fields] AND "neoplasms"[All Fields]) OR "lung neoplasms"[All Fields] OR ("pulmonary"[All Fields] AND "cancer"[All Fields]) OR "pulmonary cancer"[All Fields])) AND ((("conversion"[All Fields] OR "conversions"[All Fields]) AND ("thoracotomy"[MeSH Terms] OR "thoracotomy"[All Fields] OR "thoracotomies"[All Fields])) OR ("thoracotomy"[MeSH Terms] OR "thoracotomy"[All Fields] OR "thoracotomies"[All Fields]))
