# Supplementary figures and images for: Risk factors for conversion to thoracotomy in patients with lung cancer undergoing video-assisted thoracoscopic surgery: A meta-analysis
Source: PLoS One. 2024 Nov 15;19(11):e0313236. doi: 10.1371/journal.pone.0313236 (PMC11567592; doi:10.1371/journal.pone.0313236)

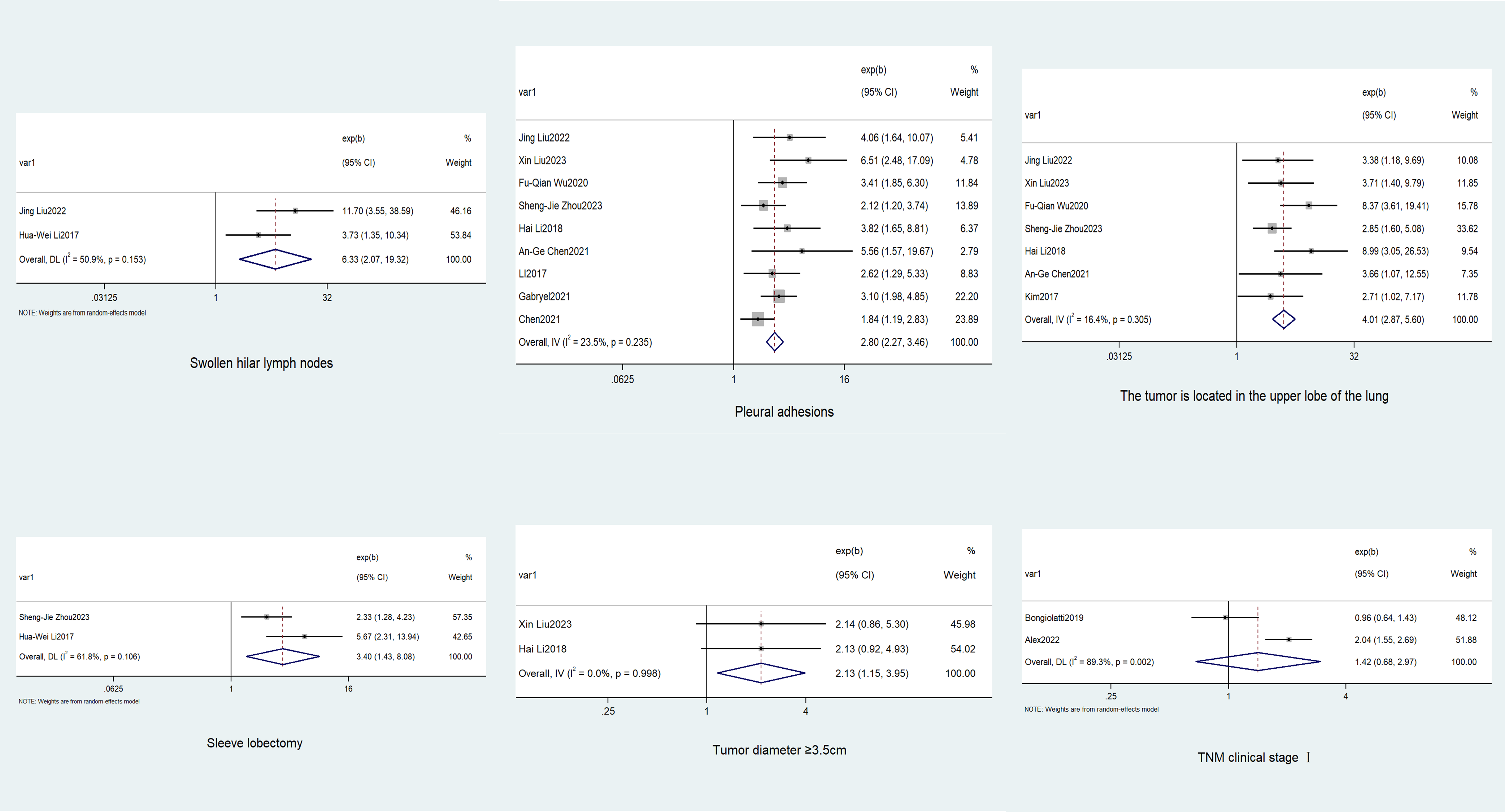

Supplement: S1 Fig — (TIF) [file pone.0313236.s005.tif]

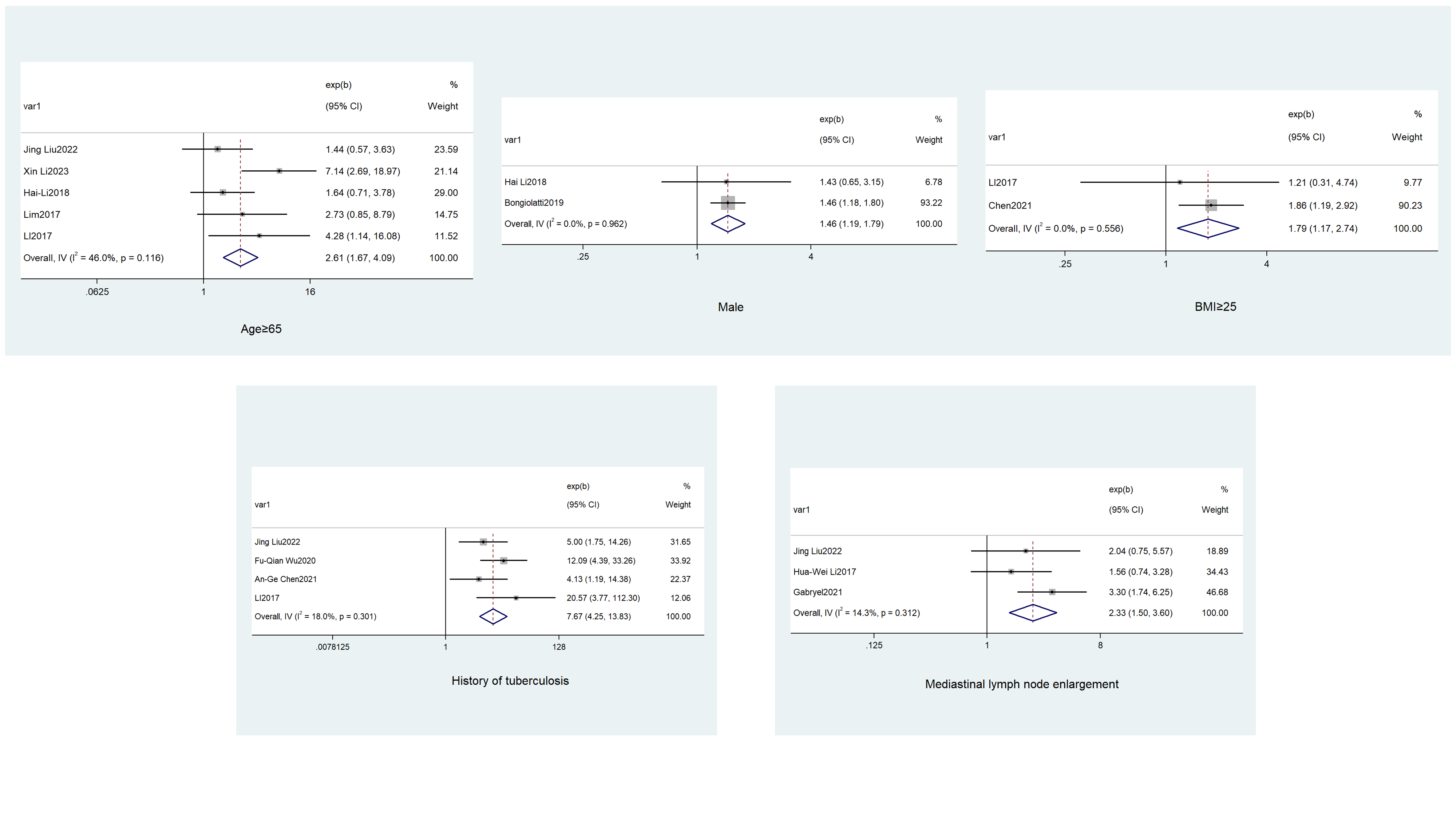

Supplement: S2 Fig — (TIF) [file pone.0313236.s006.tif]
