# Supplementary material for: Risk factors for conversion to thoracotomy in patients with lung cancer undergoing video-assisted thoracoscopic surgery: A meta-analysis
Source: PLoS One. 2024 Nov 15;19(11):e0313236. doi: 10.1371/journal.pone.0313236 (PMC11567592; doi:10.1371/journal.pone.0313236)
Supplement: Attachment — Submitted filename: Response to Reviewers.docx [file pone.0313236.s007.docx]

Dear Dr. Bertolaccini and Reviewers:

Thank you for your letter and for the reviewers’ comments concerning our manuscript entitled “Risk factors for conversion to thoracotomy in patients with lung cancer undergoing video-assisted thoracoscopic surgery: A meta-analysis” (PONE-D-24-25985). Those comments are all valuable and very helpful for revising and improving our paper, as well as the important guiding significance to our research. We have studied comments carefully and have made corrections which we hope meet with approval. Revised portions are marked in red on the paper. Furthermore, the style of the manuscript has been revised to ensure that it meets the publication requirements of PLOS ONE. All pertinent data has been collated and presented in tabular form as a supplementary document(S4). Upon closer examination, it was determined that no references to unpublished literature were identified on page 14.

We appreciate for Editors and Reviewers’ warm work earnestly, and hope that the correction will meet with approval.

Once again, thank you very much for your comments and suggestions.

Kind regards

Hong Yan.

Responds to the reviewer’s comments:

Reviewer #1:

1. In this paper, the authors considered 11 risk factors separately, also including only 2 studies on some risk factors. I think that this methodology is more suitable for a narrative review that for a meta-analysis.

Response: We are grateful for this comment. We contend that quantitative metrics offer a more objective perspective, despite the limited scope of the included literature. The risk factors for which the literature was limited were also subjected to comprehensive analysis and discussion.

2. Inclusion and exclusion criteria are not clear. It is not clear if the separate analysis were included only significant studies on the risk factor and the others did not consider it.

Response: We are very grateful for this comment. The meaning in the original article may not have been clearly expressed. All studies that included risk factors for conversion to thoracotomy during VATS will be included. The wording has been amended accordingly.

3. English should be revised.

Response: We are very grateful for this comment. The English of this article has been revised from beginning to end and hopefully will be compliant with PLOS ONE standards.

4. Discussion is very poor and it sounds as a repetition of the results, with a small contribute to clinical impact of the study.

Response: This commentary offers valuable insight that enhances the quality of this discussion. The portion of the discussion that replicated the results section was removed. Furthermore, the discussion was expanded to include the various options that physicians should consider when faced with each risk factor. For instance, patients aged 65 years and older should be operated on within 360 minutes. In cases of tumors larger than 3.5 cm, the surgeon is responsible for determining whether a thoracotomy is necessary before the operation. Similarly, patients with a history of tuberculosis must undergo imaging in advance, and the most suitable surgical procedure should be chosen based on their lymph node calcification and adhesion. Further detailed revisions are presented in the manuscript.

Special thanks to you for your good comments.

Reviewer #2:

1. The conclusion in the abstract seems quite definitive. Would it be more appropriate to qualify some statements, given the limitations you have noted?

Response: We have corrected according to the Reviewer’s comments. The abstract has included the statement ‘Due to the number and limitations of the included studies, the above conclusions need to be validated by additional high-quality studies’.

2. In the Literature Search Strategy subsection, please provide the complete search strategy for at least one database, including all search terms, Boolean operators, truncation symbols, and any limits applied.

Response: We are grateful for your proposal. The full search strategy for PubMed has been uploaded as Supplementary file S1.

3. Were any steps taken to identify unpublished or ongoing studies? This may include searching clinical trial registries, conference proceedings, or contacting experts in the field. Additionally, were any attempts made to contact study authors for missing or unclear data? Please include these details to strengthen your methodology and adhere to PRISMA guidelines.

Response: We are grateful for this question. A comprehensive search of ClinicalTrials.gov and the WHO ICTRP database was conducted, and no evidence of ongoing studies was identified. This information has been incorporated into the methodology section. Furthermore, as no data were absent from the included literature, we did not contact the authors for additional information.

4. The heterogeneity for some risk factors (e.g., hilar lymphadenopathy) is quite high. Have you considered performing subgroup analyses or meta-regression to explore potential sources of this heterogeneity?

Response: We are grateful for your counsel. The possibility of subgroup analysis has been considered in advance, but the limited availability of high-heterogeneity risk factors in only two of the included literature sources precludes their analysis. We have discussed the potential factors contributing to the high degree of heterogeneity observed, which may include the size and characteristics of the study sample, the geographical location of the research setting, and other factors.

5. The forest plots for each risk factor would be useful additions. Can these be included, perhaps as supplementary material if space is limited in the main text?

Response: We are grateful for your invaluable guidance. In consideration of the article's length, all forest plots have been provided as supplementary documentation(S3, S4).

6. Could you provide more detail on how the NOS scores influenced your interpretation of the results? For instance, did you consider giving more weight to higher-quality studies in your analysis?

We would like to express our gratitude to the reviewers for their valuable comments. The findings of the high-quality literature were largely concordant with our own, except the domain of clinical staging, where a single piece of literature offered an opposing perspective. In light of the higher NOS score awarded to the study, we believe that this is still an area worthy of further investigation. We hope that further high-quality literature will corroborate our viewpoint. This has now been incorporated into the discussion section.

7. Did you observe any temporal trends in conversion rates or risk factors when comparing earlier versus more recent studies, given the evolution of VATS techniques?

Response: This advice is beneficial for our investigation. The studies included in the article did not demonstrate any temporal trends in conversion rates or risk factors. However, a review of the literature revealed that the incidence of conversion to thoracotomy surgery during VATS decreased after 2016 in comparison to the period preceding this. This has been addressed in the discussion section.

8. Could you expand the discussion on clinical implications? For example, how might surgeons use this information to reduce conversion rates? Are there any preoperative interventions that could mitigate some of these risk factors?

Response: This commentary offers valuable insight that enhances the quality of this discussion. The portion of the discussion that replicated the results section was removed. Furthermore, the discussion was expanded to include the various options that physicians should consider when faced with each risk factor. For instance, patients aged 65 years and older should be operated on within 360 minutes. In cases of tumors larger than 3.5 cm, the surgeon is responsible for determining whether a thoracotomy is necessary before the operation. Similarly, patients with a history of tuberculosis must undergo imaging in advance, and the most suitable surgical procedure should be chosen based on their lymph node calcification and adhesion. Further detailed revisions are presented in the manuscript.

9. Consider discussing the potential impact of the observed publication bias on your results for factors where this assessment was possible.

Response: We are very grateful for this comment. The effect sizes were adjusted using the trim and fill method, and the adjusted OR was 2.50 (95% CI: 1.93-3.25), which showed no significant change compared with the pre-adjustment period. This indicates that publication bias had little effect on the results of the meta-analysis and that the results obtained were stable.

10. The manuscript would benefit from a careful review of language and grammar. For example, there are some awkward phrasings in the Results section that could be clarified.

We are very sorry for the incorrect English expression. We have revised the language section from start to finish.

Special thanks to you for your good comments.
